# Therapeutic effect of psoralen on muscle atrophy induced by tumor necrosis factor-α

**DOI:** 10.22038/IJBMS.2019.37469.8939

**Published:** 2020-02

**Authors:** Xin-Feng Lin, Qi-Long Jiang, Zhi-Long Peng, Yi-Le Ning, Yuan-Yuan Luo, Fu Zhao, Xian Peng, Wei-Tao Chen

**Affiliations:** 1 Intensive Care Unit, The First Affiliated Hospital of Guangzhou University of Chinese Medicine, Guangzhou, 510405, China; 2 Department of Spleen-Stomach, The First Affiliated Hospital of Guangzhou University of Chinese Medicine, Guangzhou, 510405, China; 3 The First Clinical School, Guangzhou University of Chinese Medicine, Guangzhou, 510405, China; 4 Lingnan Medical Research Center, Guangzhou University of Chinese Medicine, Guangzhou, 510405, China

**Keywords:** C2C12, miR-675-5P, Muscle atrophy, Psoralen, Tumor necrosis factor α

## Abstract

**Objective(s):**

To observe and determine the effect and mechanism of psoralen on tumor necrosis factor-α (TNF-α)-induced muscle atrophy.

**Materials and Methods:**

Three sets of C2C12 cells, including blank control, TNF-α (10 or 20 ng/ml) treatment and a TNF-α (10 or 20 ng/ml) plus psoralen (80 μM) administration were investigated. Cell viability was assessed using Cell Counting Kit-8 (CCK-8) assay. Western blot analysis was used to detect protein expression of atrophic markers. Flowcytometry was used to observe the effect of psoralen on apoptosis. A quantitative real-time PCR (qRT-PCR) assay was performed to detect the mRNA level of miR-675-5P.

**Results:**

TNF-α (1, 10, 20 and 100 ng/ml) treatment inhibited C2C12 myoblast viability (*P<*0.001), while 24 hr of psoralen administration increased the viability, and lowered TNF-α cytotoxicity (*P<*0.001). MURF1, MAFbx, TRIM62 and GDF15 expressions were significantly increased in TNF-α (10 ng/ml or 20 ng/ml)-treated group (*P<*0.001), and psoralen could significantly decrease the expression of these proteins (*P<*0.001). Apoptotic rate of C2C12 myoblasts was increased after TNF-α (10 ng/ml and 20 ng/ml) treatment, and was significantly decreased after psoralen treatment (*P<*0.001). miR-675-5P was increased in TNF-α-treated C2C12 myoblasts compared to control group, and it was significantly decreased after psoralen treatment.

**Conclusion:**

Psoralen could reduce TNF-α-induced cytotoxicity, atrophy and apoptosis in C2C12 myoblasts. The therapeutic effect of psoralen may be achieved by down-regulating miR-675-5P.

## Introduction

Muscle wasting is a life-threatening consequence of many critical disease (1-3) including mechanical ventilation, diabetes, chronic kidney disease and glucocorticoid-induced insulin resistance (4-6), causing the decreases of life quality and the increases of mortality. In the past decades, increased protein degradation was thought to be a primary cause of muscle atrophy. Studies have shown that by altering some genes or proteins, severe diseases can induce ubiquitin-proteasome pathways and autophagy, which play an important role in the progression of muscle atrophy (7, 8). Muscle-specific E3 ligases muscle atrophy F-box (MAFbx)/atrogin-1 and muscle ring finger-1 (MuRF1), two proteins involved in skeletal muscle protein breakdown, were found to be up-regulated in various muscle atrophy models (8-10). But, there is still too much unknown, and so far there is no effective treatment to reduce or reverse muscle atrophy. As a traditional Chinese medicine, psoralen is widely used in clinical practice. It is used alone or in combination with other Chinese herbal medicines as an anti-inflammatory drug (11), an antineoplastic drug (12), an antipyretic or an antibacterial agent. According to studies on the pharmacological effects of psoralen, we speculated that it may also has the potential to improve muscle atrophy; however, observations on the effects of psoralen on muscle atrophy were too few, and its effects and possible mechanisms are not yet clear.

Prior research has shown that microRNAs, a class of small non-coding RNAs of ~22 nucleotides, act as key regulators in the process of metabolic homoeostasis by promoting degradation or inhibiting the translation of specific mRNAs (13). It plays an important role in regulating muscle protein expression in various pathological conditions that may lead to muscle atrophy (14-16). It has been reported that miR-675-5P expressed in skeletal muscle is up-regulated during myoblast differentiation and muscle regeneration, and it could promote muscle differentiation and regeneration through negative regulation of the bone morphogenetic protein (BMP) pathway, Smad1 and Smad5 transcription factors, and DNA replication initiation factor Cdc6 (17).

Inflammatory cytokines have been shown to play a central role in the loss of skeletal muscle mass. For example, TNF-like weak apoptosis-inducing factor (TWEAK) is a major cytokine that causes muscle atrophy (18-20). In this study, we used TNF-α to treat C2C12 cells and constructed a muscle atrophy model. The effects of psoralen on cell activity and apoptosis in those muscle cells were observed, and the possible mechanism of psoralen on muscle wasting was explored.

## Materials and Methods

***Cell culture***

The myoblast (C2C12) cell line was supplied by the Cell Bank of Type Culture Collection of Chinese Academy of Sciences (Shanghai, China). Cells were incubated at 37°C in a humidified atmosphere containing 5% CO_2_ and cultured in Dulbecco’s modified Eagle’s medium (DMEM, Invitrogen) supplemented with 10% heat-inactivated fetal calf serum (Sigma Chemical Co., St. Louis, MO, USA) and 1% penicillin-streptomycin G (Invitrogen Life Technologies, Carlsbad, CA, USA). 

***CCK-8 assay***

Cell viability was determined by Cell Counting Kit (CCK-8) assay according to the manufacturer’s instructions. Briefly, C2C12 myoblasts were seeded at a density of 6×10^3^ in a 96-well plate (Corning, Costar, NY) for 24 hr. After washed by phosphate-buffered saline (PBS), we added 100 μl RPMI-1640 medium and 10 μl CCK-8 to each well. After 2 hr, the 96-well plate was measured at 450 nm using a standard micro plate reader (Scientific Multiskan MK3, thermo, USA). The cell viability was calculated according to the following formula: cell viability = (OD of the experimental sample/OD of the control group) × 100%. 

***Western blot assay***

Cells were harvested and washed with PBS and then lysed in radioimmunoprecipitation assay buffer (Beyotime, Shanghai, China). The protein concentrations were determined using a BCA Protein Assay kit (Thermo Fisher Scientific., Rockford, IL, USA). Proteins were separated using SDS-PAGE gels based on the molecular weight of the target protein and was then transferred to an PVDF membranes (PerkinElmer, Boston, MA), followed by incubation with primary antibodies overnight. The membrane was then incubated with HRP-conjugated secondary antibodies and developed using an ECL detection kit (Millipore Corp. Bedford, MA). The primary antibodies were anti-GAPDH (Dilution 1: 1000, TDY, TDY042), anti-MURF1 (Dilution 1: 1000, Abcam, ab77577), anti-MAFbx (Dilution 1: 1000, Abcam, ab168372), anti- tripartite motif containing 62 (TRIM62) (Dilution 1: 1000, Abcam, ab154635), and anti- growth differentiation factor-15 (GDF15) (Dilution 1: 1000, Abcam; ab39999).

***Flow cytometric analysis***

C2C12 myoblasts with different treatments were added into 6-wells plate and incubated overnight. Apoptosis rate was measured by flowcytometry using Annexin-V/FITC double staining (BD Biosciences, Franklin Lakes, NJ, USA) and the results were analyzed using the FlowJo software. Apoptosis of C2C12 myoblasts were analyzed in triplicates and repeated three times independently.

***RNA extraction and quantitative real-time PCR (qRT-PCR) assay***

The mRNA expression levels were detected using the SYBR-Green PCR Master Mix kit (Takara). Total RNA was isolated using TRIzol (Invitrogen, Carlsbad, CA, USA) following the manufacturer’s instructions and reversely transcribed to cDNA by RT-PCR assay using a miScript II RT Kit (Qiagen, Hilden, Germany). The target genes and controls were analyzed by qRT-PCR and the test was performed on ABI 7500 system (Applied Biosystems, Carlsbad, CA, USA) with primers specific for miR-675-5P (Qiagen, Hilden, Germany). All data are expressed as the mean±SD of three independent experiments. The sequences of primers used were as follows:

U6 primers: 

5’-CTCGCTTCGGCAGCACATATACT-3’ (forward primer) 

5’-ACGCTTCACGAATTTGCGTGTC-3’ (reverse primer); 

miR-675-5P primers: 

5’-ACACTCCAGCTGGGTGGTGCGGAGAGGGCC-3’ (forward primer) 

5’-CTCAACTGGTGTCGTGGA-3’ (reverse primer)

***Statistical analyses***

Data were analyzed by Student’s t-test and variance (ANOVA) using SPSS 19.0 software (SPSS, Chicago, IL, USA). Each experiment was repeated at least three times. Data were summarized and presented as means±SD of three or more biological replicates. *P*<0.05 was considered statistically significant. 

## Results

***Psoralen reduced TNF-*** ***α*** ***-induced cytotoxicity in C2C12 myoblasts***

**Figure 1:**
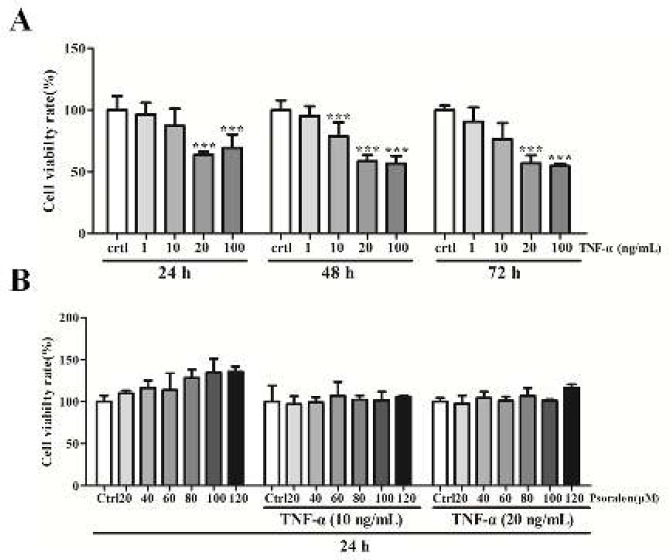
Psoralen reduced tumor necrosis factor-α (TNF-α)-induced cytotoxicity in C2C12 myoblasts. CCK-8 assay was carried out to detect cell viability. (A) C2C12 myoblasts were treated with TNF-α (1, 10, 20 and 100 ng/ml) for 24 hr, 48 hr and 72 hr, respectively. (B) Normally cultured C2C12 myoblasts and TNF-α (10 and 20 ng/ml)-treated C2C12 myoblasts were treated with psoralen (0, 20, 40, 60, 80, 100 and 120 μM) for 24 hr. ***P<0.001 vs. control (Ctrl) group

CCK-8 assay was carried out to evaluate cell viability. As illustrated in Figure 1A, treatment of C2C12 myoblasts with TNF-α (1, 10, 20 and 100 ng/ml) resulted in inhibited C2C12 myoblast viability after 24 hr, 48 hr and 72 hr of treatment (*P<*0.001), indicating that TNF-α has cytotoxic effect on C2C12 myoblasts. To examine the effect of psoralen on C2C12 myoblasts growth viability, we used different psoralen concentrations (0, 20, 40, 60, 80, 100, 120 μM) to examine on normal cultured, as well as TNF-α (10, 20 ng/ml)-treated C2C12 myoblasts. As shown in Figure 1B, after 24 hr of psoralen administration, the normal cultured C2C12 myoblast activity was increased, and the TNF-α cytotoxicity was lowered. Based on these results, we selected an intermediate concentration of psoralen (80 μM) for further study.

***Psoralen attenuated TNF-α-induced muscle atrophy***

**Figure 2:**
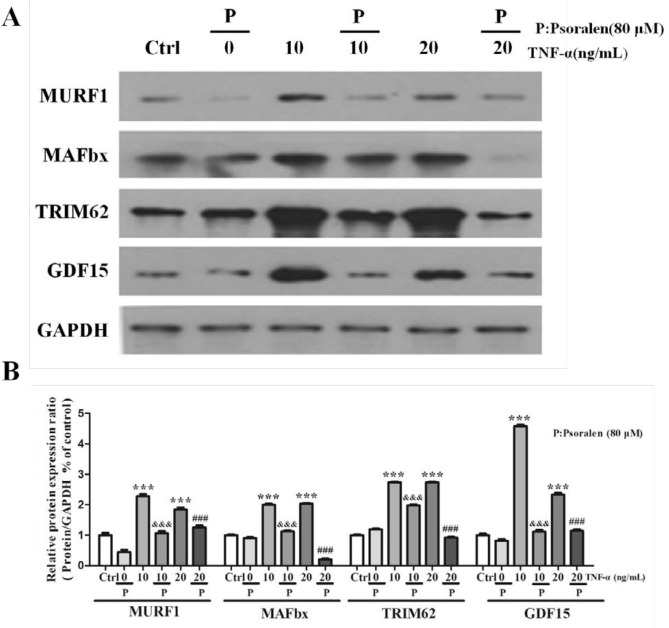
Psoralen attenuated tumor necrosis factor-α (TNF-α)-induced muscle atrophy. C2C12 myoblasts were treated with psoralen (80 μM) and TNF-α (10 and 20 ng/ml) for 24 hr. (A) Muscle ring finger-1 (MURF1), muscle atrophy F-box (MAFbx), tripartite motif containing 62 (TRIM62) and growth differentiation factor-15 (GDF15) expressions were measured by Western blot assay in C2C12 myoblasts in different groups. (B) Statistical analysis. ***P<0.001 vs. control (Ctrl) group; ^&&^*P<*0.001 vs. TNF-α (10 ng/ml) group; ^###^ *P<*0.001 vs. TNF-α (20 ng/ml) group

The protein expressions of MURF1, MAFbx, TRIM62 and GDF15 atrophy markers were measured by western blot. As showed in Figure 2, MuRF1, MAFbx, TRIM62 and GDF15 expressions were significantly increased both in 10 ng/ml group and 20 ng/ml TNF-α-treated group (*P*<0.001), and psoralen could significantly decrease the expression of these proteins (*P<*0.001).

***Psoralen inhibited TNF-α-induced apoptosis in C2C12 myoblasts***

**Figure 3:**
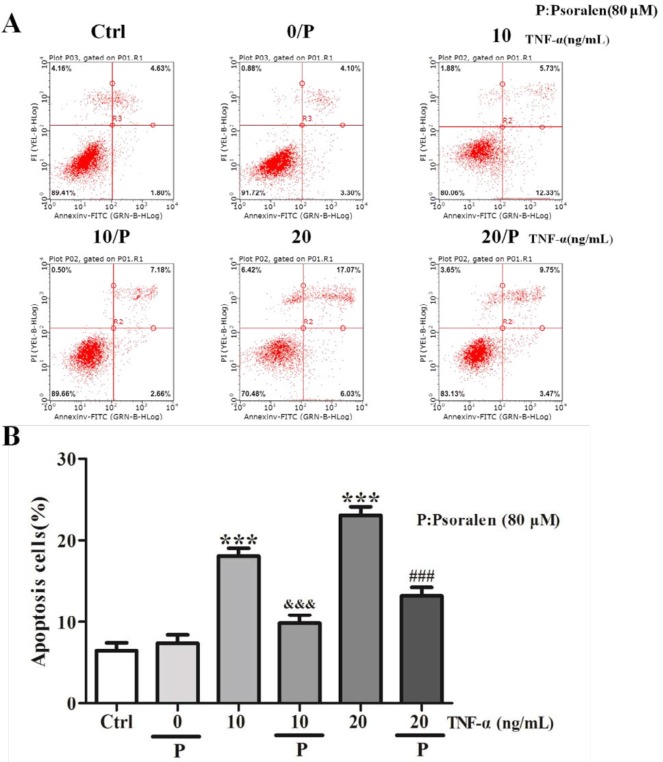
Psoralen inhibited tumor necrosis factor-α (TNF-α)-induced apoptosis in C2C12 myoblasts. C2C12 myoblasts were treated with psoralen (80 μM) and TNF-α (10 and 20 ng/ml) for 24 hr. (A) Flowcytometry result of apoptosis in C2C12 myoblasts. (B) Statistical analysis. ***P<0.001 vs. control (Ctrl) group; ^&&^*P<*0.001 vs. TNF-α (10 ng/ml) group; ^###^*P<*0.001 vs. TNF-α (20 ng/ml) group

Flowcytometry assay was used to explore whether cell apoptosis could be affected by psoralen. As showed in Figure 3, we found that the apoptotic rate of C2C12 myoblasts treated with TNF-α (10 ng/ml and 20 ng/ml) was increased compared to the control group (*P*<0.001). However, it was significantly decreased after psoralen treatment (*P*<0.001). We suggested that psoralen suppressed TNF-α-induced apoptosis in C2C12 myoblasts.

***Psoralen inhibited miR-675-5P expression in C2C12 myoblasts induced by TNF-α***

**Figure 4:**
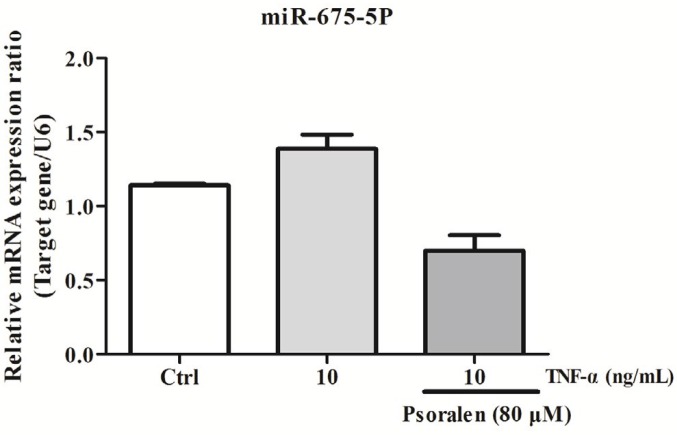
Psoralen down-regulated miR-675-5P expression in C2C12 myoblasts. C2C12 myoblasts were treated with psoralen (80 μM) and tumor necrosis factor-α (TNF-α) (10 ng/ml ) for 24 hr. qRT-PCR was used to detect the expression level of miR-675-5P in C2C12 myoblasts

The results of qRT-PCR indicated that expression of miR-675-5P was increased in TNF-α-treated C2C12 myoblasts compared to control group. However, the miR-675-5P expression was decreased after psoralen treatment (Figure 4). 

## Discussion

Muscle atrophy is caused by the imbalance between anabolic and catabolic processes. In the course of its development, the protein breakdown exceeds protein synthesis and ultimately results in a loss of muscle mass. Sepsis, cancer, diabetes mellitus, heart failure and surgery are all the reason of muscle atrophy (21-24). Exercise and myostatin silencing were reported to prevent and reverse muscle atrophy (25). 

Elevated levels of inflammatory cytokines lead to selective muscle proteins degradation (26, 27), and extracellular matrix abnormalities (28) and prevented the regeneration of skeletal muscle fibers (29, 30). Li *et al.* and Cai *et al.* demonstrated that the proinflammatory transcription factor nuclear factor-kappa B (NF-kB) regulated the expression of large number of genes associated with the ubiquitin-proteasome system (3, 31). And a series of researches showed that the loss of skeletal muscle mass induced by various catabolic stimuli (proinflammatory cytokines, tumor load, denervation, and unloading) could be attenuated by specific inhibition of the activity of NF-κB (32-34). In terms of cytokines, TNF-α has been extensively studied and is a proinflammatory cytokine known to have the most extensive role in skeletal muscle. It can impair skeletal muscle performance, accelerate muscle atrophy in critically ill patients and cause weakness (35, 36).

Accumulating evidence suggests that the activation of ubiquitin-proteasome system promoted the muscle protein degradation during the development of muscle atrophy (19, 37, 38). In 2001, a report identified that two novel E3 ubiquitin ligases, MuRF1 (Trim63) and MAFbx (FBX032), are key regulators of muscle atrophy (39). Bodine *et al.* and Gomes *et al.* confirmed that atrogin-1 or MuRF1 knockout in skeletal muscle attenuated muscle atrophy and indicated that the ubiquitin-proteasome system plays a key role in muscle wasting (39, 40). As another member of the family of E3 ubiquitin ligases, TRIM62 was identified to be involved in the regulation of differentiation, immunity, development and apoptosis, and plays an important role in Toll-like receptor 4 (TLR4) signaling (41, 42). The study performed by Langhans *et al.* showed that the expression of TRIM62 was significantly increased in the muscle of critically ill patients (43). And Trim62 activation leads to persistent inflammation in muscle and promoted atrophy in critically ill patients (44). GDF15 is known as macrophage inhibitory cytokine-1 (MIC-1) and a member of transforming growth factor beta (TGF-β) family (45). In the past few years, it has been reported that the expression level of GDF15 was elevated in various types of tumors (46, 47, 48). Recently, skeletal muscle is confirmed to secrete GDF15 in response to mitochondrial stress (49, 50). And it was identified as a secreted growth factor of aged skeletal muscle (51). 

In our study, the proinflammatory factor TNF-α (10 and 20 ng / ml) up-regulated the expression of MURF1, MAFbx, TRIM62 and GDF15 proteins in C2C12 myoblasts. In order to observe the effect of psoralen on muscle cells, we designed a cell group using psoralen alone and found that psoralen does not induce muscle atrophy in C2C12 myoblasts. However, when we used psoralen together with TNF-α in C2C12 cells, we found that psoralen could attenuate TNF-α-induced muscle atrophy. We speculate that psoralen has a protective effect on C2C12 myoblasts.

Researches show that miR-675 is overexpressed in many human cancers; however, the function of miR-675-5P is largely unknown (52). In our previous clinical study, miR-675-5P was at least 4 times up-regulated in the patients with muscle atrophy compared to healthy people (Data not shown). In this study, we attempted to detect the potential molecular mechanism. By detecting the mRNA expression level, we found that psoralen could down-regulate the expression level of miR-675-5P in TNF-α-treated C2C12 myoblasts. Therefore, we hypothesized that psoralen may protect C2C12 myoblasts by down-regulating miR-675-5P. However, unfortunately, we have not found the target of miR-675-5P, which is the direction of our next research.

## Conclusion

Overall, our current study shows that, by down-regulating miR-675-5P, psoralen can inhibit cytotoxic and pro-apoptotic effects of TNF-α-treated C2C12 myoblasts, thereby exerting its protective effect on muscle atrophy. Our research laid a clinical foundation for psoralen on the treatment of muscle atrophy, and we also provided a theoretical basis for the future research on the mechanism of muscle atrophy.
